# Interprofessional homebased reablement intervention for older adults in Sweden: a randomized controlled trial

**DOI:** 10.1186/s12877-025-05886-w

**Published:** 2025-04-10

**Authors:** Lena-Karin Gustafsson, M. Söderman, C. Johansson, M. L. Elfström

**Affiliations:** 1 Division of Caring Science, School of Health, Care and Social Welfare, Mälardalen University, Box 325, Eskilstuna, 63105 Sweden; 2 Division of Social Work, School of Health, Care and Social Welfare, Mälardalen University, Eskilstuna, Sweden; 3 Division of Psychology, School of Health, Care and Social Welfare, Mälardalen University, Eskilstuna, Sweden

**Keywords:** Home care, Home rehabilitation, Interprofessional team, Municipal home service, Older adults, RCT, Reablement, Recovery

## Abstract

**Background:**

Reablement has a health promotive perspective. The goal is to enhance or maintain health and functional ability and, thereby, the ability of older adults to live in their own homes. The intervention described in this study was introduced so the older person would remain at home and be given the opportunity to regain or maintain functional ability physically, mentally, and socially to live independently and have optimal health and well-being. This paper aims to report the measured effects of reablement among the older adults in terms of bio-psycho-social health that emerged in the randomized controlled trial (RCT).

**Methods:**

A sample of older adults (65+) was studied, consisting of those who applied for homecare in the municipal home service (*n* = 237), those who received intensive home reablement (IHR) carried out by an interprofessional team, and a control group who received home-based care as usual. Data were collected at three different occasions with validated instruments: at inclusion, after completion of IHR, and 3 months after completed intervention.

**Results:**

Both groups improved significantly at the post-measurement, and this improvement was maintained at the 3-month follow-up regarding: global quality of life (HACT); general health (EQ-5D-5 L); the self-estimates for mobility, hygiene, daily activities, pain/discomfort, anxiety/depression (EQ-5D-5 L); subjective well-being (GP-CORE); self-assessed capacity to perform physical activities as well as satisfaction with performance (COPM); measures of physical activity capacity regarding lower extremities (SPPB); upper extremities (hand dynamometer test). No between group differences were statistically significant. At the 3-month follow-up, the average number of homecare hours was slightly lower in the group that underwent IHR than in the group receiving usual homecare and rehabilitation interventions, but the difference was not statistically certain.

**Conclusions:**

In this RCT with a relatively short follow-up period, IHR was equivalent to traditional homecare regarding older people’s self-reported health, physical activity ability and number of homecare hours.

**Trial registration:**

ClinicalTrials.gov (https://clinicaltrials.gov/study/NCT03565614?intr=Reablement&rank=4) Registration number: NCT03565614. Registered on 1 January 2016.

## Background

Administering activities for recovery in daily living (ADL) functions are the main conditions for continued residence [1]. The goal of reablement is to enhance or maintain health and functional ability and, thereby, the ability of older people to live in their own homes [2–4]. Reablement includes a health promotive perspective developed from ordinary home care, aiming to maximize quality of life aspects and competencies to manage everyday life as a hole. Reablement intervention contains actions both by professionals and the person approaching an improved level of independence and consists of multiple visits by a trained and coordinated interdisciplinary team [5]. The aim of the reablement interventions is to increase or maintain independence in older adults, including those with dementia [6, 7]. It reduces the need for long-term care services [8] and is an inclusive approach regardless of age or diagnosis [9, 10]. Research shows reablement is effective but requires further studies to optimize implementation [5], as application varies between countries [11]. In Sweden, governmental agencies have shown interest in reablement promoting programmes for care in residential care [12] and in home care settings [13] but conclude that the body of scientific research on the outcomes of these programs is too scarce, and more research is needed. The current randomized control trial (RCT) was part of a larger project to highlight older persons’ experiences of the intervention and the professional team´s experiences of working with reablement [2, 3, 14, 15]. Reablement has been defined as a person-centred approach that aims to maintain or increase older adults´ chances of independence in everyday living and thereby reduce the need for long-term healthcare services [4]. Thus, in accordance with this approach, this project has attempted to integrate a person-centred approach in line with the Swedish overall ambition to implement the Good Quality, Local Healthcare Model in the field of home-based care [16]. Person-centred approach implies using a person’s values and preferences as guides for care, supporting health and the person’s own life goals [17]. Whereas integrated care involves managing and delivering healthcare services in a coordinated manner, even covering prevention tailored to the individual’s needs throughout their life [18].

The overall objective of this intervention is that the older person is reabeled in functioning ability as a predictor to remain at home and is given the opportunity to regain or maintain functional ability physically, mentally, and socially to live independently and have optimal health and well-being. In this paper, we aim to report the outcome of our three hypotheses: intensive home reablement (IHR) improves overall life satisfaction (primary), self-assessed health, health-related quality of life, and subjective well-being and physical activity capacity (secondary) and reduces the number of homecare hours (tertiary) among older adults (65+) more than traditional home care.

## Methods

This RCT carried out 2016–2020 in a middle-sized municipality in Sweden, was designed as an intervention where older adults (65+) who applied for homecare efforts in the municipal home service received IHR carried out by an interprofessional team, were compared with a control group who received home-based care and activities within ordinary social services. The protocol was drafted following the Consolidated Standards of Reporting Trials (CONSORT) guidelines [19].

### Sample

**Fig. 1:**
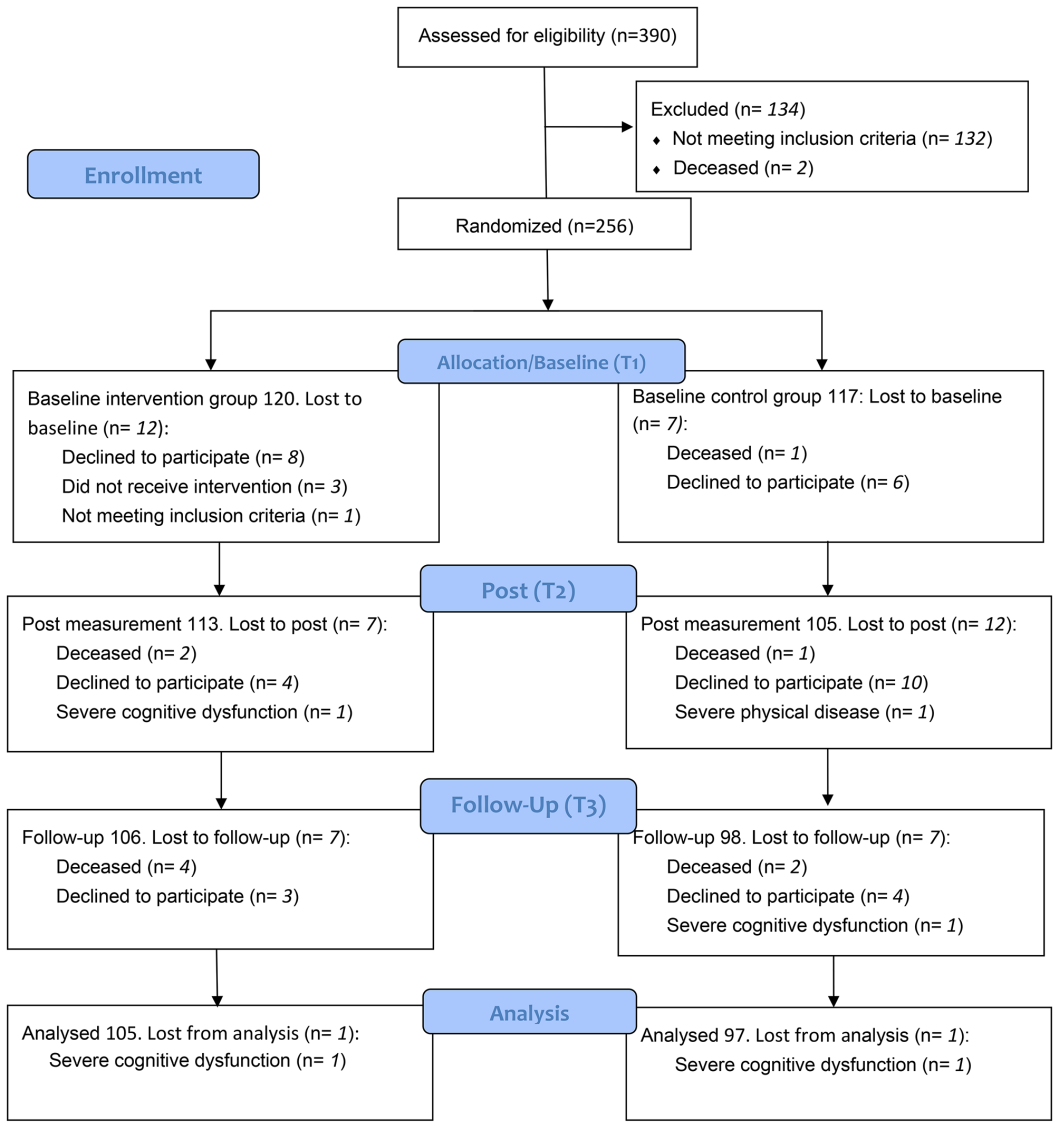
CONSORT 2010 Flow Diagram showing the process for the randomized controlled trial for the reablement intervention for older adults conducted by a multiprofessional home rehabilitation team

Prospective participants were identified by care managers when applying for homecare, and randomization to the intervention or control group was done before the question of participation was asked (Fig. 1). To have persons in both arms (intervention and active comparator/control) from the beginning of the study and use the interprofessional intervention team within the study funding limits, randomization was conducted by randomizing the order of questions of participation in blocks of four. We chose blocks of four to be more certain that we would have an equal number of participants in both groups and use the intervention team within the funded study period. Randomization was conducted using sealed envelopes with a set of two plus two outcomes, meaning that for each set two prospective participants were randomized to have the question about participation in the intervention group and two were randomized to have the question to participate in the control group. Randomization was performed by the last author who had no contact with prospective participants. The application for homecare could apply to both expansion of existing efforts as well as efforts for someone who had not had homecare before. Inclusion criteria were older adults (65 + years) who applied for municipal homecare in a middle-sized municipality in Sweden even though reablement in its form is an inclusive approach irrespective of age, capacity, diagnosis or setting [5]. Exclusion criteria were patients with severe cognitive dysfunction, life-threatening illness, serious mental illness, end of life stage or other illness/impairment that would prevent the participant from expressing their will.

### Intervention

Intensive home-based rehabilitation was carried out by an (one) interprofessional team of 21 persons (nurse, registered nurse, physiotherapist, occupational therapist, social worker) and lasted up to 3 months depending on the older person’s rehabilitation needs. The team completed a 5-week college course prior to the start of the intervention. The course was led by two of the authors of this paper (L-KG and MLE) and three other members of the initial research group. The course consisted of four weeks of full-time studies at the university in reablement as a new way of rehabilitating older people who, after a period in hospital, need homecare. The team learned theory about six overarching themes: different health perspectives, the rehabilitation process including how to set rehabilitation goals, teamwork, and motivational talks, evidence-based practice, and research participation.

The team planned for targeted rehabilitation efforts with the older person, and the care followed the decisions in collaboration with the care managers’ overall goals for accepted services. Members of the team could visit the older person’s home several times a day for rehabilitation. As far as possible, the older person met the same staff member throughout the intervention. Each participant had been allocated two contact persons with twice the time per participant as compared to home-based care and activities within ordinary municipal homecare that contain nursing (for example, support with medication, personal hygiene and supervision visits) and social service (purchasing, cleaning and laundry). The team had offices close to each other to strengthen continuous communication. At regular meetings, the interprofessional team held discussions regarding each participant, documenting outcomes in relation to rehabilitation goal activities. Mentioned goals could consist of being able go up and down the stairs, to ride the bus by yourself, being able to shower without help, etc. In the long run these goals could affect health aspects such as share bedroom upstairs with the husband, visit family and friends, upholding dignity and autonomy.

### Control group

Those randomly assigned to the control group received homecare and rehabilitation efforts according to the municipality’s previous prevailing practice. That is, assessment by aid assessors and efforts involving the same professional groups as in the intervention team depending on the assessment, following the municipality’s established routines.

### Data collection

Quantitative data were collected at three different occasions. The first, pre-measurement at baseline, in connection with inclusion (assistance assessment). There was only a couple of days or about a week between application for homecare and pre-measurement. The second, post-measurement, after completion of IHR (3 months after pre-measurement for the control group). The third, follow-up measurement, 3 months post intervention (3 months after post-measurement for the control group). People with difficulties in understanding the Swedish language in speech and writing were offered an interpreter at the information, measurement, and interview sessions.

At the pre-measurement, background characteristics was collected regarding age, gender, living situation, level of education, medical diagnoses, medication, whether care efforts were sought for the first time or if it was an extended effort, and whether an interpreter was used during the measurement. Specially trained assistants who were not involved in the participants’ rehabilitation administered the data collection in the form of questionnaires and physical tests. The number of homecare hours received was obtained via the municipality’s database at the three month follow up only.

### Measurements

Primary outcome measure, change from baseline rating at 6 months: Overall life satisfaction measured with a global rating on a horizontal visual analogue scale with end points “very dissatisfied with life” (coded 0) and “very satisfied with life” (coded 100) where higher rating indicates higher life satisfaction. This type of scale is quite common in quality of life research and this particular item was included from the Health as Ability of Acting (HACT) questionnaire which has been validated and tested for test-retest reliability with satisfactory properties [20].

Secondary outcome measures, change from baseline ratings at 6 months: Self-assessed general health was indicated by a global rating given on a vertical scale ranging from 0 (the worst health you can imagine) to 100 (the best health you can imagine) included in the EQ-5D-5 L [21]. Health-related quality of life in five domains (mobility, self-care, usual activities, pain/discomfort, anxiety/depression) was measured by the EQ-5D-5 L [21] with one 5-point Likert-scale item per domain. Higher value means more problems [22]. Subjective well-being was mirrored with a mean of 14 items comprising the General Population Clinical Outcomes in Routine Evaluation (GP-CORE). Means may range 0–4 where lower value means more well-being. The GP-CORE has showed satisfactory psychometric properties to be used to measure and monitor subjective well-being in older adults (> 65 years) in the general population of community dwelling [14, 23, 24]. Self-assessed activity performance and self-assessed activity performance satisfaction was used from the Canadian Occupational Performance Measure (COPM) [25], both rated on a 10-point scale where higher value means better performance and better satisfaction with the activity performance respectively. The COPM has support for use in research in a home-dwelling heterogeneous population of older adults [26]. Lower extremities physical activity performance was measured with the test Short Physical Performance Battery (SPPB) where results are summarized into a scale between 0 and 12, where higher scores indicate better physical function [27]. The SPPB has been shown work well in older adults and to be predictive of subsequent disability in persons over 70 years. Upper extremities physical activity performance was tested with a well-established hand dynamometer test which measured grip and hand strength reported in kilograms using the Jamar hydraulic hand dynamometer model J00105 [27]. Tertiary outcome measure was the number of homecare hours needed as recorded from the municipality database at 6 months, because previous and number of homecare hours at pre-measurement were not valid measures of homecare need.

### Statistical analyses

A pilot study of working methods, measurements and measuring instruments as well as ability to test statistical power, was carried out in autumn 2015, with a total of 25 older persons divided into an intervention group of 15 older persons and a control group of 10. The pilot study showed a medium effect size (Cohen’s d = 0.46) for global estimation of quality of life in favour of the intervention, which conservatively interpreted indicated at least 98 persons per group. There was thus a total sample of 200 persons based on alpha = 0.05 with 80% power. Consideration was given to an estimated dropout of 20% based on previous intervention studies within Swedish municipal homecare [28, 29]. Therefore, the goal was to include 240 people in the RCT.

The low proportion of incomplete data was replaced where possible (GP-CORE) with the mean value of answered statements within the relevant index, but in other cases incomplete data were not replaced. The level of significance was set at *p* < 0.05. To detect any differences in background conditions between intervention and control group, Chi-square test and Fisher’s exact text were used for categorical data, while Mann-Whitney parameter-free U-test was used for age. Non-response was studied through comparisons of pre-measurement values in those who had a complete series of measurements versus those who lacked post and/or follow-up measurements. To avoid randomly occurring significances in mass testing, a Bonferroni correction was applied to the significance levels of the 11 secondary outcome measures setting the level of significance to 0.004.

The effect analyses were performed according to “intention to treat” (ITT). The analysis method for intervention effects in primary and secondary outcome measures was 2 × 2 mixed design (group x measurement occasion) analysis of covariance (ANCOVA) with the pre-measurement of each dependent (effect) variable included as a covariate. In the few cases where problems with necessary assumptions for ANCOVA were indicated, parameter-free methods (Mann-Whitney’s U-test and Friedman’s test) were used to evaluate possible effects on the parametric analysis. For the tertiary endpoint, the groups were not comparable with respect to homecare hours at the baseline measurement due to the inclusion criteria and assessment of participants allocated to IHR. This was the case because a component of being included in the intervention group was that considerably more time with the interprofessional team was allocated to the participants, as compared to the control group where need of home care in hours was assessed using established, more conservative, routines. Hence, the intervention group per definition had more home care hours at baseline. Therefore, only their number of homecare hours at the 3-month follow-up was compared. Due to the non-normal distribution in the variable homecare hours, a parameter-free comparison method (Mann-Whitney’s U-test) was used. In some effect analyses, there were individuals with extreme values in the dependent variables, defined as Z > 3.29 and isolation from other participants’ values. In these cases, analyses were performed to evaluate the impact of the extreme values on the effect analyses.

## Results

### Group comparison at baseline

There were no statistically significant differences regarding background conditions or outcome measures between the groups at the pre-measurement (Table 1). In the intervention group, those who were lost at the post-measurement did not differ from those who completed the intervention regarding background conditions or outcome measures at the pre-measurement. In the control group, there were higher estimates of capacity to perform activities (from the COPM) among those who were lost at the post-measurement than among those who remained at the post-measurement. There was no statistically significant difference between those in the intervention group who were lost at the follow-up measurement and those who participated in this final measurement regarding background conditions and outcome measures at the pre-measurement. In the control group, there was a greater proportion of men among those who were lost to follow-up than among those who remained.

**Table 1:** Description of background conditions and measurements for the participants in RCT (*N* = 237)

Background conditions	Intervention group (IHR) *n* = 120	Control group (usual care) *n* = 117	*p* value
**Age**			0.94
Mean (standard deviation)	83.6 (7.29)	83.7 (7.40)	
Min – Max	67–99	65–99	
	**n (%)**	**n (%)**	
**Sex**			0.66
Woman	91 (76%)	85 (73%)	
Man	29 (24%)	32 (27%)	
**Educational level**			0.57
Primary/secondary school	66 (55%)	62 (53%)	
Collage/university	54 (45%)	55 (47%)	
**Co-habitant**			0.77
Yes	34 (28%)	31 (26.5%)	
No	86 (72%)	86 (73.5%)	
**Diagnosis**			0.52
Yes	110 (92%)	104 (89%)	
No	10 (8%)	13 (11%)	
**Medication (regular)**			1.00
Yes	119 (99.2%)	117 (100%)	
No	1 (0.8%)	0 (0%)	
**First health/welfare efforts**			0.10
Yes	102 (85%)	89 (76%)	
No	18 (15%)	28 (24%)	
**Interpretator**			1.00
Yes	3 (2.5%)	2 (2%)	
No	117 (97.5%)	115 (98%)	

#### Primary hypothesis

IHR improves overall life satisfaction more than traditional home care.

Both groups improved significantly at the post-measurement, and this improvement was maintained at the follow-up. At the 3-month follow-up, the groups had similar values regarding global quality of life (from HACT). No between group differences were statistically significant (Table 2).

#### Secondary hypothesis

IHR improves self-assessed health, health-related quality of life, subjective well-being, and physical activity capacity more than traditional home care.

Both groups improved significantly at the post-measurement, and this improvement was maintained at the follow-up. At the 3-month follow-up, and after a Bonferroni correction, the groups had similar values regarding self-assessed general health (from EQ-5D-5 L) the self-estimates for mobility, hygiene, daily activities, pain/discomfort, anxiety/depression (from EQ-5D-5 L), subjective well-being (from GP-CORE), self-assessed capacity to perform physical activities as well as satisfaction with performance (COPM). The same was true for the measures of physical activity capacity regarding lower extremities (SPPB) and upper extremities (hand dynamometer test). No between group differences were statistically significant (Table 2).

#### Tertiary hypothesis

IHR reduces the number of homecare hours more than traditional home care. At the 3-month follow-up, the average number of homecare hours was slightly lower in the group that underwent IHR than in the group that had usual homecare and rehabilitation interventions, but the difference was not statistically certain (Table 2).

**Table 2:** Outcome of measurements used in the RCT (*N* = 237)

**Primary Outcome Measure change from baseline rating at 6 months (3 months after completed intervention)**			
	**Intervention group** ***n*** ** = 120**	**Control group** ***n*** ** = 117**	
**Outcome**	**Mean**	**Mean**	***p*** ** value**
Overall life satisfaction	70.04	67.54	0.53
**Secondary Outcome Measures change from baseline ratings at 6 months (3 months after completed intervention)**			
	**Intervention group** ***n*** ** = 120**	**Control group** ***n*** ** = 117**	
**Outcome**	**Mean**	**Mean**	***P*** value*
Self-assessed general health	60.04	60.21	0.97
Health-related quality of life in five dimensions,			
- mobility	2.47	2.73	0.03
- self-care	1.66	1.77	0.41
- usual activities	2.45	2.70	0.09
- pain/discomfort	2.57	2.60	0.90
- anxiety/depression	1.81	1.69	0.11
Subjective well-being	1.00	1.06	0.68
Self-assessed activity performance	6.80	6.16	0.60
Self-assessed activity performance satisfaction	6.85	6.45	0.28
Lower extremities physical activity performance	4.41	4.89	0.38
Upper extremities physical activity performance	Right side 30.38 Left side 28.82	Right side 30.89 Left side 27.54	0.47 0.67
**Tertiary Outcome Measures at 6 months (3 months after completed intervention)**			
	**Intervention group** ***n*** ** = 120**	**Control group** ***n*** ** = 117**	
**Outcome**	**Mean**	**Mean**	***p*** **value**
Number of home care hours needed	30.7	33.5	0.29

NOTE *A Bonferroni correction for the secondary outcomes showed a significance level of 0.004

## Discussion

The aim of this RCT was to evaluate the effects of IHR compared to traditional care interventions by measuring multidimensional health among older people. The results showed that both the group receiving IHR and the group receiving traditional homecare improved over time in a statistically reliable manner in the outcome measures. However, there was no statistically significant difference between the groups depending on the form of rehabilitation. For the secondary outcome mobility there was a difference in favour of the control group that turned out non-significant as a Bonferroni correction to prevent Type I-errors was applied. None of our three hypotheses was thus supported, and the evidence for IHR could not be extended.

In this regard, our study adds to the body of knowledge supporting reablement as an effective and inclusive approach to reducing the need for long-term care services. Given that the application of reablement varies between countries [11] this study shows that, in the form of IHR within a Swedish home care setting, reablement is about as effective as ordinary home care services.

The results from the current RCT are similar to a previous RCT with adults with various diagnoses [30]. A 12-week home-based reablement programme with older adults [31] and a 6-week programme with patients suffering from stroke, had similar effects regarding the control group on patients’ perceived performance [1].

Regarding effectiveness, interventions that employ comprehensive effectiveness in geriatric care assessment are often weak due to lack of statistical power [32]. Methodologically, the current RCT appears to be reliable, as it followed established procedures without crucial deviations. For example, the randomization procedure worked well, as the groups were comparable at baseline, and a large enough number of people participated in the study to achieve statistical power in the calculations. The procedure of randomization before allocation, before the participants were invited, can be seen as a limitation. However, the aid assessors were clear that it is difficult for older persons to consent if it is not possible to describe what the participants were to consent to. Most of the older persons were in a vulnerable situation and, according to the aid assessors, possibly having to change homecare staff would be a concern for many of them. The non-attendance analyses do not indicate any systematic bias affecting the efficacy measure behind the participants who were lost after the pre-measurement. The self-assessment instruments used (EQ_5D-5 L, GP-CORE, COPM) have all been shown to be sensitive to change in different ways, which is also the case with the global estimates of general health (EQ_5D-5 L) and life satisfaction (HACT), while the physical tests (SPPB, hand dynamometer) are well established in this type of study. Regarding the post-measurement after completion of IHR (3 months after pre-measurement for the control group) there can be a limitation in that the second measurement could differ. However, most participants in the intervention group needed 3 months intervention time, meaning that this measurement point was similar in both groups.

However, this project gives no information about what happens in the longer term as the follow-up period was quite short for all effect measures and results. A nearby supplementary remark about the importance of follow-up is made in the sub-study about older peoples’ experiences of a rehabilitation process regarding the potential importance of follow-up contacts for the continuation of various rehabilitation exercises [15]. Specifically for the impact measure homecare hours, it can be pointed out that it is a single indicator of care and care needs and does not give any idea of the situation when transitioning to other forms of housing than one’s own housing. Forms of housing and other more direct measures of the economical result of the intervention remains to be studied. Furthermore, as an inclusion criterion was applying for home care (first time or increased number of hours) the previous number of home care hours were not a valid measure of home care need. Depending on which group the participants were allocated to, the assessment of home care hours at inclusion were different between the two groups and consequently a comparison of home care hours at inclusion would not be valid. Thus, the test of the tertiary hypothesis was only performed for homecare hours at 6 months and is more a comparison than an evaluation.

The intervention’s validity (fidelity) was supported by the entire team completing a 5-week joint university course and was monitored by each older person having a contact person with double the time for the older person compared to usual homecare, regular team meetings regarding all older persons receiving IHR and that all individual goals and efforts was documented. On the other hand, this is a complex intervention that was delivered by several professionals. It is thus difficult to fully guarantee that all participants received the intended intervention in all parts, as indicated by the sub-study about older peoples’ experiences of a rehabilitation process regarding challenges in formulating individual rehabilitation goals [15]. Although active knowledge transfer or information provision between the IHR homecare-group and the municipality’s regular homecare-groups were avoided, it cannot be excluded that the knowledge that the IHR intervention was taking place may have influenced regular homecare-groups to some extent.

Besides knowledge transfer, there is also another aspect of transfer between the intervention team and the researchers in that two of the present paper authors led the training course proceeding the intervention, with three other members of the initial group of researchers. Thus, there is a risk that researchers’ interest in achieving certain results could have influenced the team. To prevent such influence the researchers were not involved in the interprofessional team´s work with the older persons. The researchers and the interprofessional team belonged to different organizations, had different offices and no contact about the intervention as it was carried out. The interprofessional team worked out their own working procedures, based on the content of the training course. Furthermore, neither the researchers nor the intervention team were practically involved in the data collection at the three measurement points, as specially trained assistants who were not involved in the participants’ rehabilitation administered the data collection in the form of questionnaires and physical tests.

An alternative view of the statistical equivalence between the groups in the RCT is that the IHR resulted in a higher level of demand on the participants than for those who had traditional homecare. The alternative view is that, despite the higher level of demand, the older persons receiving IHR had as good self-rated health and quality of life as those who had traditional homecare. The sub-study results about older persons’ perceptions of caring skills in the short-term goal-oriented rehabilitation process, illustrate a range of possible supportive aspects of the IHR team that may have contributed to the health and quality of life of the older people in the intervention group [3]. Examples of these supporting aspects can be found in the qualitative studies, where, among other things, the treatment and the relationship between the older persons receiving IHR and the IHR team are highlighted as important for successful intervention [2, 3]. In addition, the interviewees believe that individual rehabilitation goals, preferably with social claims and continuous support from both relatives and neighbours, affect the sustainability over time in their own housing [15].

### Conclusions and relevance for practice

In the RCT with a relatively short follow-up period, IHR was equivalent to traditional homecare regarding older people’s self-reported health, physical activity ability and number of homecare hours. As implication for praxis, it becomes interesting for further research to see how specific differences in background factors might affect the reablement outcomes to be able to refine the intervention and thereby tailor the best conditions for remaining in own homes.

## Data Availability

The data cannot be shared openly, to protect the study participants privacy. The datasets generated and analysed during the current study are not publicly available due to limitations of ethical approval involving the patient data and anonymity but are available from the corresponding author on reasonable request.

## Appendix 1: Overview of measurements used in the RCT

**Table:**

**Primary Outcome Measure change from baseline rating at 6 months**	
**Outcome**	**Instrument**
Overall life satisfaction	Horizontal visual analogue scale from 0 to 100 where higher rating means higher life satisfaction, reported in Health as Ability of Acting questionnaire (HACT) (Snellman et al. 2011).
**Secondary Outcome Measures change from baseline ratings at 6 months**	
**Outcome**	**Instrument**
Self-assessed general health	Global rating given on a vertical scale ranging from 0 (the worst health you can imagine) to 100 (the best health you can imagine) included in the EQ-5D-5 L (Herdman et al., 2011).
Health-related quality of life in five domains,	One 5-point Likert item per domain where higher value means more problems in the domain, items are included from the EQ-5D-5 L (Herdman et al., 2011).
- mobility	
- self-care	
- usual activities	
- pain/discomfort	
- anxiety/depression	
Subjective well-being	5-point mean of 14 items from the General Population Clinical Outcomes in Routine Evaluation (GP-CORE). Means may range 0–4 where lower value means more well-being (Hochwälder et al., 2022, Evans, et al., 2002).
Self-assessed activity performance	10-point scale where higher value means better self-assessed activity performance, measured with Canadian Occupational Performance Measure (COPM) (Law et al., 1990).
Self-assessed activity performance satisfaction	10-point scale where higher value means more satisfaction with the activity performance, measured with Canadian Occupational Performance Measure (COPM) (Law et al., 1990).
Lower extremities physical activity performance	Scale between 0 and 12 that summarizes the test Short Physical Performance Battery (SPPB) (Guralnik et al., 1994), where higher scores indicate better physical function.
Upper extremities physical activity performance	A hand dynamometer test measured with grip and hand strength reported in kilograms (Mathiowetz et al., 1985) using the Jamar hydraulic hand dynamometer model J00105.
**Tertiary Outcome Measures change from baseline ratings at 6 months**	
**Outcome**	**Instrument**
Number of home care hours needed	Municipality database

## Abbreviations

ADL: Activities in daily living
CONSORT: Consolidated Standards of Reporting Trials
DPA: The Swedish Data Protection Authority
GDPR: General Data Protection Regulation
IHR: Intensive home reablement
RCT: Randomized controlled trial
